# Chicago Dental Society

**Published:** 1884-06

**Authors:** A. E. Baldwin


### CHICAGO DENTAL SOCIETY.

### MAY MEETING.

REPORTED FOR THE INDEPENDENT PRACTITIONER BY A. E. BALDWIN, M. D.

President Harlan in the chair.

The subject of the evening was "Correction of Irregularities."

The first thing was an excellent, though brief paper on the subject, by Dr. J. G. Reid, who said it was a subject upon which much had been written and that many plans had been advocated. Of all the great variety of operations demanded of the dentist none should be given more scrupulous thought, care and attention than this. Teeth when in proper relation and proper shape articulate perfectly, and in such cases mastication is thoroughly performed. He desired to impress upon all the necessity of a study of the character of the deformity, avoiding haste. No two cases are exactly alike in cause or result. Without the earnest co-operation of the patient, treatment will probably end in failure.

The first thing necessary is to get a *perfect* impression of the parts, and of the many ways to accomplish this he thought that with plaster by far the best. He believes that black rubber is as a rule the best for regulating plates. It is easily adapted, cheap, and non-irritant. Believes Dr. Coffin's plan of spreading the arch is the best, but the dentist should select wire of a size suitable to the age of patient. Often small pieces of sea-tangle tent may be used with advantage to wedge between plate and teeth. These pieces should be changed once in two or three days. As a rule, he was not in favor of the use of jack-screws. Finds more difficulty in regulating the lower than the upper arch. He often does it by first spreading the upper with Coffin's plate, then retaining, and thus, by throwing the force of mastication on the outer cusps of the lower teeth, moves them out. Thinks rubber bands are of great service in almost any condition, but in the use of them care is required to keep them from irritating the gums. They may sometimes be used by slitting the band part way round, letting the split portion rest on the crown of the tooth. Sometimes they may be retained by small pluglets of cotton between the teeth. While much may be said against the plan, he yet believes that many cases may be facilitated by judicious extraction. His paper was accompanied by many models and appliances.

*Dr. Crouse*—Commended the paper highly for its radicalness, and that was a characteristic necessary to the accomplishment of anything in any field of labor. You must insist upon the co-operation of the patient. The plate should be worn in the mouth instead of in the pocket. The earlier you begin in life the better. Thought modeling compound the best material for taking impressions. Thinks Coffin plates are useful, but as a rule would prefer jack-screws, as they spread the arch more rapidly. *Be sure your plates are worn.* Regulating should be made a specialty, and the man who attends to this should do nothing else. Regarding extracting, he thinks cases requiring it are very rare. Believes more failures result from not retaining the teeth in position after moving them than from any other cause. You should insist on cleanliness. In answer to a question, Dr. Crouse said that in his hands Dr. Patrick's method had not proved as satisfactory as he could have wished.

*Dr. Talbot*—Spoke of Dr. Farrar's and Dr. Patrick's methods, but said he thought they would not meet all requirements. Is a firm believer in the jack-screw and wedge, with their many modified forms, as the simplest and easiest methods of moving teeth.

*Dr. Noyes*—Suggested that the lower arch could be easily spread by securing a model and cutting it in two in the mesial line, then spreading the heel or molar end of it slightly, imbedding it in plaster, making a plate to fit, and springing this on the teeth, the spring of the plate producing the required pressure.

*Dr. Brophy*—Enlarged upon the necessity for studying each case. In order to retain the teeth in place after moving them, he often uses a modification of Dr. Patrick's apparatus. A thin platinum band is fitted around a molar or bicuspid on each side, and a platinum bar attached to one band and passed through a loop in the other. The teeth are fastened to this bar; or platinum bands may be fitted to the teeth, and then united to a light gold or platinum band inside the arch.

*Dr. Newkirk*—Said he had come to like modeling compound better than plaster for impressions. He thinks the reason so many disliked it was because they did not let it get cold enough before removal. He suggested holding a sponge wet with cold water against it in the mouth; or a lump of ice may be used. Believes the successful regulator is the one who is mechanician enough to improvise the necessary apparatus for the special case.

*Mrs. Dr. Mann*—Said that in getting sea-tangle tent one should examine and select only the pieces that are solid, as many are hollow. She condemns the rubber bands and kindred appliances, as they produce constant pressure, and thus awaken inflammation. Thought the jack-screw method was preferable, as after each removal of the teeth a period of rest would follow, thus preventing inflammatory action.

*Dr. Harlan*—Would not commend any particular method. Much had been said about what to do. He would speak of something one ought *not* to do, and that is extract lateral incisors or cuspids under any circumstances. The dentist should use judgment, patience, and common sense. Crowded and irregular arches are often caused by not retaining the temporary cuspids, though a large majority of cases arise from such extractions. In using Coffin plates he recommended loosening the spring gradually. Thinks these plates are better than jack-screws. In constructing retaining plates he makes the edge of the plate next the teeth of soft rubber.

*Dr. Crouse*—Spoke of a band with bar attached as an excellent appliance to use in moving individual teeth. Fasten the band on with oxy-phosphate. In answer to a question how he retains teeth in position after regulating, said he often made a light, thin, skeleton plate of celluloid.

*Dr. Noyes*—Recommended Dr. Black's plan for retaining, by moving the teeth with figure of eight ligatures.

*Dr. Swain*—Spoke of a couple of cases of peculiar deformity that he had treated, in which he had capped the molars until the bicuspids had come up in line; then capped the bicuspids until the molars came up.

The Society adjourned to meet the first Tuesday in June.

